# Carotenoid and Phenolic Compositions and Antioxidant Activity of 23 Cultivars of Corn Grain and Corn Husk Extract

**DOI:** 10.3390/foods13213375

**Published:** 2024-10-23

**Authors:** Shaokai Cai, Yuxiang Mao, Yongjian Gu, Bowen Huang, Zhiyong He, Maomao Zeng, Zhaojun Wang, Qiuming Chen, Mingxia Tang, Jie Chen

**Affiliations:** 1 School of Food Science and Technology, Jiangnan University, Wuxi 214122, China; skycaisk@163.com (S.C.); zyhe@jiangnan.edu.cn (Z.H.); mmzeng@jiangnan.edu.cn (M.Z.); zhaojun.wang@jiangnan.edu.cn (Z.W.); chenqm@jiangnan.edu.cn (Q.C.); 2 State Key Laboratory of Food Science and Resources, Jiangnan University, Wuxi 214122, China; 3 Institute of Agricultural Sciences of Jiangsu Changjiang River Bank District, Nantong 226012, China; yxmao6901@163.com (Y.M.); gyj1071734460@sina.com (Y.G.); 4 Wuxi Food Safety Inspection and Test Center, Wuxi 214142, China; 15161518560@163.com; 5 Technology Innovation Center of Special Food for State Market Regulation, Wuxi 214142, China

**Keywords:** corn, lutein, polyphenols, flavonoids, antioxidant activity

## Abstract

As a byproduct of corn processing, corn husk is usually burned or disposed of. To make a better use of corn husk, its bioactive components need to be further explored. In this work, the carotenoids and phenolics of the extracts from the corn grain and corn husk of 15 different yellow corn and 8 different waxy corn were identified and quantified, and their antioxidant activities were assessed. The results showed many considerable variations in carotenoid contents. Four types of carotenoids were observed only in both yellow corn and black waxy corn. The highest lutein and zeaxanthin contents were both observed in yellow corn husks. Lutein dominates in yellow corn, ranging from 494.5 μg/g dw to 2870.8 μg/g dw, which is followed by zeaxanthin, ranging from 63.0 μg/g dw to 360.2 μg/g dw, and finally β-cryptoxanthin and β-carotene. The total content of polyphenols (TPC) and flavonoids (TFC) of the husk from 13 yellow corn cultivars, as well as the TPC of husk from 8 waxy corn cultivars, were all higher than those of their corn grain, with the highest TPC found in waxy corn husk. Additionally, a total of 20 phenolic compounds were identified, and ferulic acid showed the highest content and reached 1101.9 µg/g dw in a waxy corn husk. The average antioxidant activity of a waxy corn husk was 25–65% higher than that of a yellow corn husk, and the highest values were observed in the husk of the waxy corn cultivar Huhong 1. These results suggested that corn husk is a rich source of lutein and phenolics and provided excellent cultivars as a reference for functional food products in agriculture and the food industry.

## 1. Introduction

Corn, the third most important crop after rice and wheat, is available in many parts of the world [1]. As the world’s second-largest consumer of corn, China maintains a stable planting area of over 40 million hectares. Corn grains are a rich source of nutrients, containing corn starch, protein, and dietary fiber. Because of their content of phenolic compounds and carotenoids, corn grains are known to possess higher antioxidant activity than wheat, oat, and rice grains. It is suitably used as an industrial material, playing a significant role in agricultural production [2]. Corn husk, covering the corn core and accounting for about 15% of the corn kernel [3], primarily consists of cellulose, lignin, and other polysaccharides. It is an important by-product of the corn processing industry, usually used in animal feed or fermentation industry, and also offers pharmacological effects such as lipid-lowering, blood pressure reduction, and intestinal disease prevention [4,5,6]. Previous research on corn husk mainly focused on the extraction of dietary fibers and polysaccharides [7], but there are still many active ingredients, such as carotenoids or phenolics, yet to be fully explored and utilized.

Carotenoids are divided into two subclasses: hydrocarbon carotenoids and oxygen-containing xanthophylls. The lutein class includes lutein (C_40_H_56_O_2_), zeaxanthin (C_40_H_56_O_2_), cryptoxanthin, astaxanthin, capsanthin, and fucoxanthin, among others. Carotenoids have various isomers such as α, β, and γ-carotene, among which β-carotene has the strongest physiological activity [8]. Pigments extracted from yellow corn primarily consist of lutein, zeaxanthin, β-cryptoxanthin (C_40_H_56_O), and β-carotene (C_40_H_56_) [9], which can serve as natural food colorants. As the only two types of macular pigment (MP) in the retina, lutein and zeaxanthin can protect ocular tissues from blue light and monoclinic oxygen radicals and alleviate retinal macular degeneration and cataracts [10]. Additionally, they have nutritional functions such as immune regulation, cancer cell proliferation inhibition, and arteriosclerosis delay [11]. However, lutein can not be synthesized in the human body and must be ingested through dietary intake [12], so the increasing research has focused on lutein extraction recently. Lutein-rich food resources mainly include green vegetables, marigolds, and corn. Previously, there were some reports on the comparisons of lutein extraction from different cultivars of corn [13] and the separation and determination of lutein using high-performance liquid chromatography (HPLC) methods [14]. However, there have been no detailed studies about the yellow pigments between the corn grain and corn husk of the same cultivar.

Polyphenols are also one of the significant active components of corn husk. As secondary metabolites in plants, polyphenols include phenolic acids, flavonoids, coumarins, tannins, and stilbenes [15]. They can offer beneficial effects such as free radical scavenging, antioxidant, anti-inflammatory, antitumor, antibacterial properties, and immune enhancement [16]. The edible part of the grain contains fewer polyphenols, while most polyphenols are concentrated in the bran of the grain [17]. In recent years, researchers have increasingly focused on the identification and bioactivity of polyphenols from grains. Wen et al. [18,19] conducted a comprehensive study on the metabolome of corn kernels. In the study, a secondary mass spectrometry labeling (MS2T) database and a corn kernel flavonoid metabolic network were established, and 39 flavonoid substances, including naringenin, apigenin, vitexin, and hesperidin, etc., were identified and annotated. Lau et al. [20] optimized the method of enzyme-assisted extraction of ferulic acid from sweet corn cob, Chen et al. [21] had detected 13, 12, 12, and 8 types of polyphenols from corn kernel, bract, core, and silk except for corn husk of Nongtian 88 sweet corn, respectively, Shalini et al. [22] had determined phenolic substances and their antioxidant activity of white, yellow, and purple corn. Previous studies have mostly focused on the determination of corn polyphenols and corn silk polyphenols using HPLC and HPLC-MS. However, there are few studies have been conducted to accurately characterize and quantify polyphenols in different cultivars of corn husk.

Therefore, we expect to obtain more information about bioactive compounds and functional properties of different corn, which could lay a good foundation for developing whole grain foods and nutraceuticals or food ingredients based on corn. This study aims to characterize the carotenoids and phenolics extracted from 15 cultivars of yellow corn and 8 cultivars of waxy corn. And the antioxidant activity of corn extracts was evaluated by 2,2′-azinobis (3-ethylbenzothiazoline-6-sulfonic acid) (ABTS), 1,1-diphenyl-2-picrylhydrazyl (DPPH) scavenging activity, and ferric reducing antioxidant power (FRAP). To our knowledge, this was the first study to report a comprehensive characteristic of the carotenoids and phenolic extracts of grain and husk of different corn cultivars. The research is critical to enable consumers to gain greater access to the health benefits of corn. This may encourage local growers and industries to recognize the potential of corn husks and make full use of them instead of discarding them as waste, which would be of great significance for the promotion of their benefits.

## 2. Materials and Methods

### 2.1. Chemicals

All of the carotenoid standards (lutein, zeaxanthin, β-cryptoxanthin, and β-carotene) with purity of ≥90% were purchased from YuanYe Biotechnology Co. Ltd. (Shanghai, China). Both of the phenolic compound standards (naringenin, ferulic acid, isofraxidin, luteolin-7-glucoside, cyanidin-3-glucoside, isoquercitin, quercetin, and rutin) with purity of ≥96% were purchased from J&K Scientific Co., Ltd. (Beijing, China). Folin-Ciocalteu reagent, 2,2′-azinobis (3-ethylbenzothiazoline-6-sulfonic acid) (ABTS), 1,1-diphenyl-2-picrylhydrazyl (DPPH), 2,4,6-tripyridyl-s-triazine (TPTZ), and 6-hydroxy-2,5,7,8-tetramethylchroman-2-carboxylic acid (Trolox) were supplied by Sigma-Aldrich Chemical Co. (St. Louis, MO, USA). HPLC-grade methanol, acetonitrile, and tert-butyl methyl ether (MTBE) were purchased from TEDIA Company, Inc. (Fairfield, OH, USA). The 95% ethanol and other solvents and reagents used in this study were of analytical grade and purchased from Sinopharm Chemical Reagent Co., Ltd. (Shanghai, China).

### 2.2. Plant Material and Pretreatment

**Figure 1:**
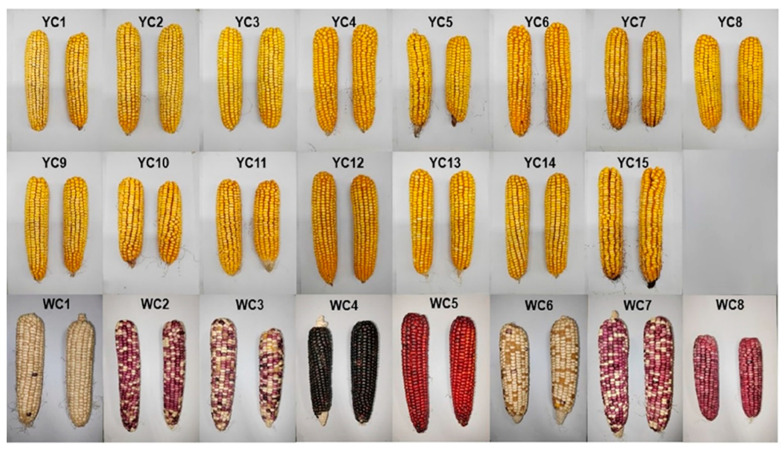
23 cultivars of corn (YC1–YC15 are 15 cultivars of yellow corn and WC1–WC8 are 8 cultivars of waxy corn).

Different corn cultivars were provided by Jiangsu Yanjiang Institute of Agricultural Sciences (Nantong, Jiangsu, China), including 15 cultivars of yellow corn and 8 cultivars of waxy corn, named ‘Sukeyu 1’, ‘Sukeyu 2’, ‘Huai’, ‘Jiangyu 1’, ‘Jiangyu 2’, ‘Zhongjiang 1’, ‘Zhongjiang 2’, ‘Tongyu 1’, ‘Tongyu 2’, ‘Tongyu 3’, ‘Tongyu 4’, ‘Tongyu 5’, ‘Suyu’, ‘Tongyu 6’, ‘Tongyu 7’ for yellow corn, labeled YC1 to YC15, and ‘Wan’, ‘Suke’, ‘Suyu 1’, ‘Huzihei 2’, ‘Huhong 1’, ‘Sutian 1’, ‘Suyu 2’, ‘Suyuzi’ for waxy corn, labeled WC1 to WC8. The corn grain and corn husk were ground and sieved through an 80 mesh screen and stored at −20 °C in the dark. Photos of the 23 cultivars of corn are shown in Figure 1.

### 2.3. Extraction of Lutein and Polyphenol

Lutein and phenolic compounds from corn were extracted according to the method reported by Ahmad et al. [23] with some modifications. The dried 1 g of corn grain or corn husk powder was added into 15 mL 95% ethanol and extracted on a magnetic stirrer (IKA RT 10, Guangzhou, China) at 40 °C in the dark for 1 h. The supernatants were collected after centrifugation at 8000 rpm for 10 min at room temperature, and the solid residue was extracted one more time under the same conditions. The two supernatants were combined and volume to 30 mL, then stored at −20 °C under N_2_ in the dark until analysis.

### 2.4. Separation and Quantification of Carotenoids

The extract solutions above were filtered through a 0.22 μm micropore membrane into amber glass vials before HPLC analysis. Carotenoids were separated and quantified according to the method described by Moros et al. [24] with slight modifications. A Waters e2695 high-performance liquid chromatograph equipped with a 2998 PDA detector (Waters Corporation, Milford, MA, USA) (HPLC-PDA) was used to quantify the carotenoids in corn extractions. The column was a Waters YMC Carotenoid S-5 column (250 mm × 4.6 mm, 5 μm). The column temperature was 30 °C. The injection volume of the prepared sample was 20 μL. The detection wavelength was 446 nm. Solvent A was acetonitrile/methanol (70:30), and solvent B was MTBE. The solvent flow rate was 1 mL/min, and the gradient was as follows: 0–12 min, 100–80% A; 12–17 min, 80–0% A; 17–17.1 min, 0–100% A; 17.1–20 min, 100% A; 20–22 min, 100–0% A; 22–22.1 min, 0–100% A; 22.1–25 min, 100% A. Identification of the main carotenoids compounds was performed by comparing the retention time of peaks with external standards, and the levels of lutein, zeaxanthin, β-cryptoxanthin, and β-carotene were calculated from their linear calibration curves of the corresponding standard and expressed as μg/g of the dry weight (dw) of corn grain or husk.

### 2.5. Determination of Total Phenolic Content

The total phenolic content in the extracts was determined using the Folin-Ciocalteu method adapted from Cheng et al. [25]. The 1 mL of 0.2 M Folin-Ciocalteu reagent was mixed with 0.25 mL of the sample. Then, 3 mL of Na_2_CO_3_ solution (75 g/L) and distilled water were added, and the final volume was brought to 10 mL. After incubation for 2 h at room temperature in the dark, the absorbance was measured at 765 nm using a microplate reader (SpectraMax 190, Molecular Devices, CA, USA). Quantification was based on the standard curve established with gallic acid and expressed as gallic acid equivalents in milligrams per gram dry weight of corn (mg GAE/g dw).

### 2.6. Determination of Total Flavonoid Content

The total flavonoid content in the extracts was estimated by colorimetric assay [26]. The 2.0 mL of the sample was supplemented with distilled water to a final volume of 4 mL, followed by the sequential addition of 0.3 mL of 5% NaNO_2_ solution. After incubation for 5 min, 0.3 mL of 10% AlCl_3_ solution was mixed and allowed to react for 6 min. Then, 2 mL of 1 M/L NaOH solution was added. The volume was finally brought to 10 mL and reacted for 10 min at room temperature. The absorbance of the solution was measured at 510 nm using a microplate reader. Rutin was used as the standard, and the results were expressed as milligrams of rutin equivalents (RE) per gram dry weight of corn powder (mg RE/g dw) based on the standard curve.

### 2.7. Identification and Quantification of Polyphenols

The extracts were analyzed using the X500R high-resolution mass spectrometry system (AB SCIEX, Framingham, MA, USA), equipped with a UHPLC system (AB SCIEX, USA) and OS data acquisition software (AB SCIEX, USA). MS-DIAL ver.5.1.230912 software and the MSMS_Public_ExpBioInsilico_NEG_VS17.msp mass spectrometry database were used for spectral library searching.

Chromatography conditions were as follows. For positive ion mode, the chromatographic column was Waters ACQUITY UPLC^®^ BEH C8 (1.7 μm, 2.1 × 100 mm), the column temperature was 50 °C, and the injection volume was 4 μL. Solvent A was 0.1% formic acid in water, solvent B was 0.1% formic acid in acetonitrile, the flow rate was 0.3 mL/min, the gradient elution program was as follows: 0–2 min, 5% B; 37–42 min, 5–99% B; 42.1–45 min, 100%B; returning to 5%B. For negative ion mode, the chromatographic column was Waters ACQUITY UPLC^®^ HSS T3 (1.8 μm, 2.1 × 100 mm), the column temperature was 50 °C, and the injection volume was 4 μL. Solvent A was 6.5 mM ammonium bicarbonate in water, solvent B was 6.5 mM ammonium bicarbonate in 90% methanol/water, the flow rate was 0.3 mL/min, and the gradient elution program was similar to the positive mode.

Mass spectrometry conditions included MS full scan and IDA secondary ion scan. For positive ions, source temperature was 550 °C, curtain gas flow was 35 psi, declustering potential (DP) was 80 V, collision energy (CE) was 10 eV, MS/MS collision voltage was 40 ± 15 eV, primary MS scan range 70–1050 Da, secondary MS scan range 50–1050 Da. For negative ions, source temperature was 350 °C, curtain gas flow was 35 psi, declustering potential (DP) was −80 V, collision energy (CE) was −10 eV, MS/MS collision voltage was 35 ± 15 eV, primary MS scan range 70–1050 Da, secondary MS scan range 50–1050 Da.

Polyphenol compounds were identified by using SCIEX OS ver.1.5.0.23389 software, MS DIAL software, and the MSMS-Public-ExpBioInsilico-VS17.msp mass spectrometry database and MSMS-MetaboBASE, along with reference literature. The content of naringenin, ferulic acid, isofraxidin, luteolin-7-glucoside, cyanidin-3-glucoside, isoquercitin, quercetin, and rutin was quantified by external standard method, and calculated using regression equations from the standard curves, whereas other polyphenols were quantified as ferulic acid because of the insufficiency of the standard. Concentrations were expressed as μg/g of the dry weight (dw) of corn powder. The μg/g dw^1^ meant the polyphenols were quantified as standard, and μg/g dw^2^ meant it was quantified as ferulic acid.

### 2.8. Determination of Antioxidant Activity

DPPH free radical scavenging activity was determined using the method described by Sarikurkcu et al. [27] with slightly modified. DPPH ethanol solution (2 mM/L) and Trolox standards (0–100 μM/L) were prepared. The 100 μM/L Trolox standard solution was diluted to 80, 60, 40, 20, and 10 μM/L. The 100 μL of 0.2 mM/L DPPH ethanol solution and an equal volume of standard solution were added to a 96-well plate and reacted for 30 min at room temperature in the dark. Absorbance at 517 nm was measured, and the anhydrous ethanol was used as the blank control. The Trolox standard curve was established. The DPPH scavenging activity of the samples was expressed as the Trolox equivalent antioxidant capacity (TEAC) in micromoles Trolox per gram dry weight of corn powder (μM TE/g dw).

ABTS radical scavenging activity was detected according to the method of Quan et al. [28] with modifications. ABTS (7 mM/L) and potassium persulfate (2.45 mM/L) were mixed and left at room temperature in the dark for 14 h to prepare the ABTS radical stock solution. Before use, the stock solution was diluted with phosphate-buffered saline (PBS, pH 7.4, 0.2 M/L) to achieve an absorbance of 0.700 (±0.020) at 734 nm. Trolox standards (0–1000 μM/L) were prepared. The 1000 μM/L Trolox standard solution was diluted to 800, 600, 400, 200, and 100 μM/L. The 10 μL of the standard solution was added along with 190 μL of the ABTS working solution to a 96-well plate. The reaction was conducted for 10 min in the dark at room temperature, and the absorbance at 734 nm was measured with PBS as the blank. The ABTS scavenging activity of the samples was expressed as the Trolox equivalent antioxidant capacity (TEAC) in micromoles Trolox per gram dry weight of corn powder (μM TE/g dw).

FRAP test was performed according to the method described by Qie et al. [29] with modifications. TPTZ stock solution (10 mM/L dissolved in 40 mM/L HCl), FeCl_3_ solution (20 mM/L), and acetate buffer (0.3 M/L, pH 3.6) were mixed in a ratio of 1:1:10 and allowed to rest for 1 h at 37 °C to prepare the fresh FRAP working solution. Trolox was used to establish the standard curve. Trolox stock solution (1000 μM/L) was prepared and then diluted to concentrations of 800, 600, 400, 200, 100, and 50 μM/L to make the standard solutions. The 10 μL of standard solution and 190 μL of FRAP working solution were added to a 96-well plate and reacted for 30 min in the dark at room temperature. The absorbance at 593 nm was measured with distilled water as the blank. The FRAP value of the samples was obtained by replacing the standard solution with the samples and was expressed as the Trolox equivalent antioxidant capacity (TEAC) in micromoles Trolox per gram dry weight of corn powder (μM TE/g dw).

### 2.9. Statistical Analysis

All data were performed in triplicate, and they were presented as mean ± standard deviation. Data were analyzed using Statistix 9.0 software for one-way ANOVA and performed to determine significant differences (*p* < 0.05). All the tests were used to compare the means.

## 3. Results and Discussion

### 3.1. Carotenoid Compositions in the Different Cultivars of Corn

The main carotenoid compounds such as lutein, zeaxanthin, β-cryptoxanthin, and β-carotene were detected, and some peaks in the HPLC chromatogram have not been identified because some standards are unavailable. The quantitative results of the main carotenoid compounds extracted from 23 cultivars of corn grain and corn husk are listed in Table 1. The carotenoids could only be detected in the yellow corn and black waxy corn, while they were undetectable in other waxy corn. The most predominant carotenoid was lutein, followed by zeaxanthin, finally β-carotene and β-cryptoxanthin.

Lutein is one of the common xanthophyll carotenoids in nature [24]. It has a polyene chain with ten conjugated double bonds, which functions as a chromophore and displays the characteristic yellow-red color. The 15 cultivars of yellow corn showed considerable differences in their content of lutein. For corn grain, the three cultivars with the highest lutein content were Tongyu 4 (2438.18 μg/g dw), Tongyu 2 (2259.93 μg/g dw), and Tongyu 5 (2219.63 μg/g dw), while the lowest lutein content was observed in Tongyu 7 (494.54 μg/g dw) and Tongyu 6 (588.67 μg/g dw). For corn husk, the highest lutein contents were found in the cultivars Tongyu 4 (2870.76 μg/g dw) and Tongyu 2 (2870.76 μg/g dw), and the content was lowest in Tongyu 3 (456.12 μg/g dw) and Tongyu 6 (509.21 μg/g dw). The data showed the highest lutein content of the extracts was about 6 times the lowest values. Furthermore, the lutein concentration difference between corn grain and corn husk varied widely among the different cultivars, ranging from 30 μg/g dw of the cultivar Suyu to about 740 μg/g dw of the cultivar Zhongjiang 1. Among the 8 cultivars of waxy corn, lutein was detected only in Huzihei 2, while the content in corn grain and husk were both less than 150 μg/g dw and rather similar. This data showed that the extraction of lutein from waxy corn was much lower than that of yellow corn, and it was in agreement with the previous research, which confirmed that the main pigments in purple, red, or black corn are anthocyanin [30].

Zeaxanthin always exists with lutein. Results showed that the average zeaxanthin content was approximately 10% of the lutein content, and the average value was 130 μg/g dw in corn grain and 150 μg/g dw in husk. For corn grain, the cultivars with the highest zeaxanthin content were Tongyu 3 (313.96 μg/g dw) and Zhongjiang 1 (265.56 μg/g dw), while the contents were lowest in Huai (63.01 μg/g dw) and Suyu (68.04 μg/g dw). For corn husk, the highest zeaxanthin contents were observed in the cultivar Tongyu 3 (360.17 μg/g dw) and Tongyu 7 (243.76 μg/g dw), and the lowest were in Tongyu 6 (75.83 μg/g dw) and Zhongjiang 2 (78.13 μg/g dw). Besides, the amount of zeaxanthin between corn grain and husk was similar in the cultivar Tongyu 6, and the highest difference value was 110 μg/g dw in Zhongjiang 1 and Tongyu 7. These results are in agreement with the previous research that the main carotenoid in corn is lutein, which is much more than zeaxanthin [31].

β-cryptoxanthin is one of the lutein. The cultivars with the highest β-cryptoxanthin content in corn grain were Tongyu 1 (2.14 μg/g dw) and Jiangyu 1 (2.06 μg/g dw), while the values were lower than 2 μg/g dw in the remaining cultivars. For corn husk, the highest β-cryptoxanthin content was in Jiangyu 1 with 0.90 μg/g dw. Among the four carotenoids analyzed, β-cryptoxanthin amount was the lowest, and its average value was only 0.1% of the lutein. Additionally, the highest β-carotene contents of corn grain were detected in the cultivars Tongyu 1 (13.03 μg/g dw) and Tongyu 3 (12.67 μg/g dw), while the highest β-carotene of corn husk was found in Tongyu 3 (9.66 μg/g dw) and Tongyu 1 (9.36 μg/g dw). The average β-carotene extraction amounts in corn grain and husk were about 8 μg/g dw and 6 μg/g dw, respectively, which was about 0.4% to 0.6% of the average lutein content.

In this study, the high levels of lutein and zeaxanthin indicate that they are the major components of yellow corn pigment extracts, and their variations in content between the different cultivars of corn grain and husk were presented. The research indicates that the discarded corn husks contain high levels of lutein, suggesting their potential to be an alternative raw material for lutein supplementation and lutein-enriched functional foods development. This also provides valuable insights for selecting excellent corn husk for lutein extraction and offers guidance for variety selection and cultivation in agricultural production.

### 3.2. TPC and TFC in the Extract of Different Corn Grain and Husk

Table 2 presents the TPC and TFC of the ethanol extracts of the 23 cultivars of corn grain and husk. For the corn grain, the average TPC (1.2650 mg GAE/g dw) in waxy corn was slightly higher than that (1.0703 mg GAE/g dw) in yellow corn, and the average TFC (1.0626 mg RE/g dw) was 34% higher than that (0.7924 mg RE/g dw) in yellow corn. The highest TPC and TFC of yellow corn were both found in the cultivar Suyu, with 1.2814 mg GAE/g dw and 1.2058 mg RE/g dw in the extracts, respectively. The highest TPC (1.5803 mg GAE/g dw) and TFC (1.9007 mg RE/g dw) of waxy corn were respectively found in Huhong 1 and Huzihei 2, which were both higher than the highest values in yellow corn. For the corn husk, the average TPC (2.3981 mg GAE/g dw) in waxy corn husk was 45% higher than that (1.6550 mg GAE/g dw) in yellow corn husk, while the average TFC (1.1330 mg RE/g dw) was slightly lower than that (1.2778 mg RE/g dw) of yellow corn husk. In addition, Tongyu 2 had the highest TPC (2.6195 mg GAE/g dw) and TFC (2.2492 mg RE/g dw) value among the yellow corn husks. Among the waxy corn husks, the highest TPC (2.8930 mg GAE/g dw) was found in Suyu 1, and the highest TFC (2.1808 mg RE/g dw) was in Huhong 1. The results showed that the average TPC value of the husk of the waxy corn and yellow corn were 90% and 55% higher than the corresponding corn grain, respectively, for the same cultivar, and the average TFC were 7% and 60% higher than the corn grain, respectively. This suggests that polyphenols and flavonoids may be more concentrated in the husk, resulting in significantly higher TPC and TFC in corn husk compared to corn grain. The result is in agreement with previous research [32] that the distribution of polyphenols and flavonoids is mainly in the pericarp. These data confirm the fact that the corn husk is an important material for the extraction of polyphenolic antioxidants, especially the cultivars Suyu 1 and Tongyu 2, which are appropriate for the utilization of polyphenols and flavonoids.

### 3.3. Qualitative Analysis of Polyphenols in the Different Cultivars of Corn

The main polyphenols of the extracts of 23 cultivars of corn grain and corn husk were identified based on the UHPLC-MS and corresponding databases. Preliminary identifications revealed 20 phenolic compounds, including 6 flavonols, 1 dihydroflavonoids, 2 biflavonoids, 2 flavanones, 4 cinnamic acids, 3 anthocyanin and 2 coumarins [33]. The retention time of the chromatographic peaks, mass spectra fragments, molecular formulas, and compound assumptions are presented in Table 3. Ferulic acid was identified and observed in the yellow corn cultivars of Sukeyu 1, Sukeyu 2, Huai, Jiangyu 1, Jiangyu 2, Zhongjiang 1 and the waxy corn cultivars of Suyu 1, Huzihei 2, Huhong 1 and Sutian, which was consisted with the previous research that it is the major non-anthocyanin polyphenolic compound in corn [34]. For yellow corn cultivars, 3-hydroxycinnamic acid was found in Sukeyu 2 and Tongyu 3, cyanidin-3,5-di-O-glucoside chloride was found in Sukeyu 2 and Jiangyu 1, quercetin-7-rhamnoside was found in the yellow corn cultivars Zhongjiang 2, Tongyu 1 and Tongyu 3 and P-coumaric acid was found in Jiangyu 2. For waxy corn, luteolin-7-glucoside, cyanidin-3-glucoside, apigenin-7-O-glucosiderutin, isoquercitin, and dihydroluteolin were only observed in the black corn Huzihei 2, which was an agreement with the previous research about anthocyanin profile of blue corn [35]. For other common flavonoids found in the corn, quercitrin was identified in Jiangyu 1 and Huzihei 2, naringenin was identified in Zhongjiang 1, Tongyu 6 and Huzihei 2, hesperetin was only identified in Tongyu 3, coumarin was identified in 2 different yellow corn cultivars Sukeyu 2 and Jiangyu 2 and 8 waxy corn cultivars, and coumaric acid was observed in Zhongjiang 1, Tongyu 2, Tongyu 4 and Suyu [36]. Isofraxidin was identified as coumarin, which was observed in all 8 waxy corn and 12 yellow corn. Finally, procyanidin A1 or procyanidin A2 were observed in all 23 corn cultivars. There were 12 types of polyphenols identified in black waxy corn Huzihei 2, which was the cultivar with the most types of polyphenols detected. Besides, no more than 6 types of polyphenols were detected in other cultivars. Additionally, there were many biological functions of the main polyphenols. Ferulic acid has the functions of regulating lipid metabolism, anti-oxidation as well as anti-inflammation [34]. Procyanidin could achieve health functionality by improving the intestinal environment [35], and other polyphenols, such as naringenin, have strong antioxidant activity.

### 3.4. Quantification of Polyphenols in the Different Cultivars of Corn

The main polyphenol contents in the extracts of 23 cultivars of corn grain and corn husk are shown in Table 4 and Table 5, respectively. For yellow corn, the highest content (333.79 μg/g dw^1^) of ferulic acid was found in the corn grain of Suke 1, which was higher than that in its husk. For waxy corn, the content of ferulic acid was the highest in the corn grain (736.98 μg/g dw^1^) and corn husk (1101.90 μg/g dw^1^) of Huhong 1. Isofraxidin was observed in most corn cultivars. The highest content of isofraxidin was found in corn grain (89.98 μg/g dw^1^) and husk (70.52 μg/g dw^1^) of Zhongjiang 1 among yellow corn, and it was the highest in corn husk (75.58 μg/g dw^1^) of Huhong 1 among waxy corn. Some polyphenols were observed in many cultivars and quantified as ferulic acid; others were detected in a few cultivars. The black waxy corn Huzihei 2 was found to contain a variety of polyphenols. It was observed that there were naringenin (25.99 μg/g dw^1^), luteolin-7-glucoside (425.94 μg/g dw^1^), cyanidin-3-glucoside (172.16 μg/g dw^1^), isoquercitin (4.38 μg/g dw^1^), apigenin-7-O-glucosideand (461.81 μg/g dw^2^) and dihydroluteolin (118.07 μg/g dw^2^) in the extracts of corn grain of Huzihei 2. Its extract of corn husk was also observed containing rutin (0.80 μg/g dw^1^), quercetin (14.48 μg/g dw^1^), and dihydroluteolin (170.48 μg/g dw^2^). For other corn grains, 3-hydroxycinnamic acid was observed in the Sukeyu 2 (105.34 μg/g dw^2^), cyanidin-3,5-di-O-glucoside chloride and quercitrin were observed in the Jiangyu 1, and the contents were 607.70 μg/g dw^2^ and 1.14 μg/g dw^1^, respectively. For corn husk, 3-hydroxycinnamic acid and hesperetin were found in the Tongyu 3 and the contents were 80.68 μg/g dw^2^ and 57.24 μg/g dw^2^, respectively, cyanidin-3,5-di-O-glucoside chloride was found in the Sukeyu 2 (698.30 μg/g dw^2^), and p-coumaric acid was found in Jiangyu 2 (42.52 μg/g dw^2^).

### 3.5. Antioxidant Activity of Extract from Different Corn Grain and Husk

In general, lutein and phenolic or flavonoid compounds possess some degree of antioxidant activity. Therefore, the extracts with a higher lutein or phenolic and flavonoid content generally show higher antioxidant activity. The antioxidant activity of the different corn grains and husks was measured by DPPH, ABTS, and FRAP assays, which are shown in Table 6.

Among the extracts of yellow corn, the highest DPPH radical scavenging activity was found in the cultivar of Suyu, with 2.00 μM TE/g dw in corn grain and 1.87 μM TE/g dw in corn husk, respectively. Among the waxy corn extracts, the grain of black corn Huzihei 2 and the husk of red corn Huhong 1 had the highest DPPH value at 3.01 μM TE/g dw and 2.32 μM TE/g dw, respectively. Besides, the average DPPH scavenging activity of the extracts of waxy corn grain (1.90 μM TE/g dw) and corn husk (1.52 μM TE/g dw) were 30% and 25% higher than that of yellow corn, respectively. The result is consistent with previous research that the DPPH radical scavenging activity in black corn was highest, followed by white and yellow waxy corn at the same maturity stage [53]. For yellow corn, the highest ABTS scavenging activity of corn grain and husk was observed in the cultivar Tongyu 6 (13.57 μM TE/g dw) and Zhongjiang 2 (19.03 μM TE/g dw), respectively. For waxy corn, the highest ABTS values of corn grain and husk were found in Huzihei 2 (9.88 μM TE/g dw) and Huhong 1 (26.57 μM TE/g dw), respectively. Besides, the average values of the extracts of waxy corn husk (18.83 μM TE/g dw) and yellow corn husk (14.36 μM TE/g dw) were 152% and 124% higher than the values of corn grain, respectively. For yellow corn, the cultivar of Tongyu 6 had the highest FRAP of 3.82 μM TE/g dw among corn grains, and Tongyu 1 had the highest value of 5.05 μM TE/g dw among corn husks. For waxy corn, the highest value of corn grain and husk were found in the cultivar of Huzihei 2 (5.53 μM TE/g dw) and Huhong 1 (8.12 μM TE/g dw), respectively. Additionally, the average FRAP of waxy corn husk (5.18 μM TE/g dw) and yellow corn husk (3.14 μM TE/g dw) were 45% and 27% higher than the values of corn grain, respectively. Other studies have reported the antioxidant properties of colored corn [54] and the excellent TEAC values of waxy corn [55] or rice [56]. Additionally, a report on azuki beans indicated that almost all the seed coat extracts showed substantially powerful antioxidant capacity, but the extracts from their cotyledons almost none [57].

To further explore the influence of different compounds on the antioxidant activity of corn extracts, a correlation analysis between antioxidant activity and lutein content, TPC, and TFC was carried out, as shown in Table 7. The regression analysis showed good correlations (R^2^) between TPC or TFC and these three antioxidant activity values with regression coefficients close to 0.90 in the extracts of waxy corn husk, but no significant correlation between lutein and antioxidant activity values was observed, which indicated that the high content of polyphenols and flavonoid may be the major contributors to the antioxidant activity of the waxy corn husk. This is consistent with the research that revealed the significant correlation between TEAC values and TPC and TFC in Marigold [58]. However, a significant correlation between lutein content and DPPH, ABTS, and FRAP values was not found in yellow corn grain, which showed a negative correlation. Additionally, there was no significant correlation between the lutein content, TPC, or TFC and the three antioxidant activity values in yellow corn husk. Other studies revealed that antioxidant activity is usually concentration-dependent of single polyphenols or lutein [59], but the combinations of polyphenols and carotenoids may have synergistic or antagonistic effects depending on their types and concentrations. There may be a stronger antioxidant formation between the polyphenol and carotenoid due to regeneration or complexation in the yellow corn [60], which enhances or weakens the antioxidant activity of the system when concentrations reach certain levels in yellow extracts [61]. Furthermore, these data indicated that the content of lutein, TPC, or TFC may not be the main contributor to the yellow corn. The results appeared to suggest that other compounds in the corn extracts may also be attributed to the antioxidant activity, such as ω-6/ω-3 polyunsaturated fatty acids (PUFAS) [62] or alkaloids [63], which could be co-extracted with lutein and had the high reducing power.

## 4. Conclusions

The current study showed that variations of up to several-fold in the carotenoid and phenolic content existed in different cultivars of corn, and the waste corn husk is a natural source of lutein and phenolic, which could be explored further to fully utilize. HPLC analysis confirmed that the carotenoid compositions in corn grain and husk were similar in 15 cultivars of yellow corn, and lutein dominates, followed by zeaxanthin and low concentrations of β-carotene and β-cryptoxanthin. The highest lutein and zeaxanthin were both observed in yellow corn husks. Only Huzihei 2 showed detectable levels of carotenoids among 8 cultivars of waxy corn, which is much lower than yellow corn. Besides, the average TPC and TFC values in waxy corn were higher than that of yellow corn, and the average contents in corn husk were higher than in corn grain. A total of 20 polyphenols were identified from the 23 cultivars of corn, and ferulic acid was the major polyphenol. The different cultivars of corn also showed considerable variations in their antioxidant activities. The antioxidant activity of corn husk was higher than that of corn grain, and the correlation between the DPPH, ABTS, and FRAP values of waxy corn husk extracts and TPC or TFC was significant. In conclusion, the corn husk of the cultivar Tongyu 2 was found to have the highest lutein content, as well as the highest phenolic and flavonoid contents, and therefore is a promising candidate for the production of corn extracts. The research provides a valuable reference for the comprehensive utilization of corn husk and seed selection of the corn, which contribute to the enhancement of added value of agricultural production.

## Figures and Tables

**Table 1:** The contents of four types of carotenoids in extracts of different cultivars of corn grain and husk.

NO.	Sample	Lutein (μg/g dw)		Zeaxanthin (μg/g dw)		β-Cryptoxanthin (μg/g dw)		β-Carotene (μg/g dw)	
		Corn Grain	Corn Husk	Corn Grain	Corn Husk	Corn Grain	Corn Husk	Corn Grain	Corn Husk
YC1	Sukeyu 1	620.34 ± 7.31 ^k^	903.48 ± 2.46 ^i^	86.59 ± 3.24 ^i^	169.99 ± 5.65 ^de^	0.83 ± 0.02 ^fg^	0.53 ± 0.02 ^cd^	5.62 ± 0.02 ^k^	6.73 ± 0.02 ^f^
YC2	Sukeyu 2	1338.20 ± 11.45 ^h^	1191.13 ± 7.87 ^h^	123.78 ± 2.68 _e_	135.67 ± 4.01 ^g^	1.09 ± 0.03 ^d^	0.62 ± 0.01 ^b^	7.70 ± 0.14 ^h^	5.98 ± 0.06 ^g^
YC3	Huai	1817.87 ± 12.64 ^e^	1753.78 ± 2.67 ^d^	63.01 ± 2.13 ^k^	98.31 ± 2.34 ^i^	0.47 ± 0.05 ^i^	0.31 ± 0.01 ^h^	3.93 ± 0.02 ^l^	4.81 ± 0.04 ^i^
YC4	Jiangyu 1	806.08 ± 18.44 ^i^	675.76 ± 4.07 ^m^	111.05 ± 0.74 ^f^	93.13 ± 2.22 ^i^	2.06 ± 0.04 ^b^	0.90 ± 0.05 ^a^	12.27 ± 0.05 ^c^	7.57 ± 0.05 ^e^
YC5	Jiangyu 2	1719.58 ± 13.84 ^f^	2043.91 ± 6.30 ^c^	93.13 ± 2.22 ^h^	102.04 ± 4.98 ^i^	0.86 ± 0.04 ^f^	0.48 ± 0.02 ^de^	8.07 ± 0.05 ^g^	4.42 ± 0.02 ^j^
YC6	Zhongjiang 1	1495.43 ± 10.91 ^g^	755.07 ± 3.59 ^k^	265.56 ± 3.93 ^b^	156.02 ± 4.26 ^f^	1.27 ± 0.01 ^c^	0.38 ± 0.02 ^fg^	10.40 ± 0.28 ^d^	4.49 ± 0.06 ^j^
YC7	Zhongjiang 2	1509.71 ± 6.86 ^g^	1348.95 ± 6.33 ^g^	105.97 ± 2.81 ^g^	78.13 ± 2.21 ^jk^	1.09 ± 0.02 ^d^	0.43 ± 0.02 ^ef^	7.66 ± 0.11 ^h^	3.77 ± 0.05 ^k^
YC8	Tongyu 1	1954.34 ± 10.14 ^d^	1676.55 ± 4.63 ^e^	131.56 ± 1.10 ^d^	123.28 ± 2.32 ^h^	2.14 ± 0.02 ^a^	0.46 ± 0.04 ^e^	13.03 ± 0.16 ^a^	9.36 ± 0.11 ^b^
YC9	Tongyu 2	2259.93 ± 7.02 ^b^	2870.76 ± 7.61 ^a^	138.54 ± 2.50 ^c^	215.96 ± 4.22 ^c^	0.86 ± 0.04 ^f^	0.53 ± 0.02 ^cd^	8.38 ± 0.12 ^f^	8.58 ± 0.05 ^c^
YC10	Tongyu 3	676.90 ± 11.95 ^j^	456.12 ± 4.33 ^o^	313.96 ± 2.80 ^a^	360.17 ± 3.66 ^a^	1.00 ± 0.07 ^e^	0.54 ± 0.03 ^cd^	12.67 ± 0.12 ^b^	9.66 ± 0.04 ^a^
YC11	Tongyu 4	2438.18 ± 12.85 ^a^	2669.42 ± 6.66 ^b^	111.22 ± 0.87 ^f^	175.63 ± 3.98 ^d^	0.89 ± 0.03 ^f^	0.56 ± 0.04 ^c^	8.12 ± 0.08 ^fg^	6.11 ± 0.08 ^g^
YC12	Tongyu 5	2219.63 ± 13.88 ^c^	1481.60 ± 8.20 ^f^	119.79 ± 3.39 ^e^	126.40 ± 4.52 ^h^	1.24 ± 0.03 ^c^	0.61 ± 0.01 ^b^	7.24 ± 0.03 ^i^	5.62 ± 0.08 ^h^
YC13	Suyu	671.00 ± 7.78 ^j^	705.15 ± 3.64 ^l^	68.04 ± 2.15 ^k^	83.24 ± 2.29 ^j^	1.16 ± 0.05 ^d^	0.65 ± 0.04 ^b^	10.47 ± 0.05 ^d^	8.68 ± 0.05 ^c^
YC14	Tongyu 6	588.67 ± 13.20 ^l^	509.21 ± 6.51 ^n^	76.92 ± 1.36 ^j^	75.83 ± 4.12 ^k^	0.60 ± 0.07 ^h^	0.31 ± 0.01 ^h^	7.09 ± 0.06 ^i^	4.48 ± 0.06 ^j^
YC15	Tongyu 7	494.54 ± 3.21 ^m^	870.42 ± 7.37 ^j^	132.26 ± 3.01 ^d^	243.76 ± 2.66 ^b^	0.89 ± 0.03 ^f^	0.48 ± 0.02 ^de^	6.53 ± 0.09 ^j^	5.82 ± 0.22 ^h^
WC1	Wan	ND	ND	ND	ND	ND	ND	ND	ND
WC2	Suke	ND	ND	ND	ND	ND	ND	ND	ND
WC3	Suyu 1	ND	ND	ND	ND	ND	ND	ND	ND
WC4	Huzihei 2	125.45 ± 3.85 ^n^	102.00 ± 2.83 ^p^	109.81 ± 3.40 ^fg^	161.30 ± 0.92 ^ef^	0.74 ± 0.01 ^g^	0.38 ± 0.03 ^gh^	8.87 ± 0.12 ^e^	8.08 ± 0.06 ^d^
WC5	Huhong 1	ND	ND	ND	ND	ND	ND	ND	ND
WC6	Sutian	ND	ND	ND	ND	ND	ND	ND	ND
WC7	Suyu 2	ND	ND	ND	ND	ND	ND	ND	ND
WC8	Suyuzi	ND	ND	ND	ND	ND	ND	ND	ND

Data represent mean values ± standard deviations (n = 3). Different lowercase letters within the same column indicate significant differences between samples (*p* < 0.05). ND: not detected.

**Table 2:** Total phenols and total flavonoid contents of extracts from different cultivars of corn grain and corn husk.

NO.	Sample	TPC (mg GAE/g dw)		TFC (mg RE/g dw)	
		Corn Grain	Corn Husk	Corn Grain	Corn Husk
YC1	Sukeyu 1	1.0592 ± 0.0402 ^hi^	1.3211 ± 0.0212 ^m^	0.8749 ± 0.1885 ^def^	1.8809 ± 0.2862 ^b^
YC2	Sukeyu 2	0.9124 ± 0.0243 ^j^	1.6846 ± 0.0194 ^k^	0.8944 ± 0.2913 ^fghi^	0.9968 ± 0.0454 ^ghi^
YC3	Huai	0.9295 ± 0.0186 ^j^	1.9565 ± 0.0285 ^g^	0.7536 ± 0.1934 ^fghi^	1.5889 ± 0.1637 ^c^
YC4	Jiangyu 1	0.9549 ± 0.0497 ^j^	0.8369 ± 0.0287 ^p^	0.4661 ± 0.0673 ^ij^	0.5947 ± 0.0588 ^jk^
YC5	Jiangyu 2	1.0368 ± 0.0030 ^i^	2.0323 ± 0.0639 ^f^	0.8221 ± 0.2135 ^fghi^	1.3640 ± 0.0951 ^cde^
YC6	Zhongjiang 1	1.0498 ± 0.0293 ^i^	1.2269 ± 0.0604 ^n^	0.6652 ± 0.2543 ^ghij^	1.4811 ± 0.1394 ^cd^
YC7	Zhongjiang 2	1.2225 ± 0.0372 ^ef^	1.1418 ± 0.0326 ^o^	0.7814 ± 0.1061 ^fgh^	1.3609 ± 0.0803 ^def^
YC8	Tongyu 1	1.0745 ± 0.0434 ^hi^	1.4213 ± 0.0171 ^l^	0.8716 ± 0.1533 ^cdef^	2.0843 ± 0.4110 ^ab^
YC9	Tongyu 2	1.1529 ± 0.0184 ^g^	2.6195 ± 0.0249 ^c^	0.9439 ± 0.2242 ^cdef^	2.2492 ± 0.2482 ^a^
YC10	Tongyu 3	1.1529 ± 0.0844 ^fg^	1.9040 ± 0.1717 ^i^	0.8390 ± 0.1008 ^fgh^	0.8552 ± 0.1120 ^hij^
YC11	Tongyu 4	0.9679 ± 0.0157 ^j^	2.0491 ± 0.0277 ^f^	0.8691 ± 0.1550 ^fgh^	1.3399 ± 0.2339 ^cd^
YC12	Tongyu 5	1.0321 ± 0.0311 ^i^	1.8617 ± 0.0515 ^h^	1.1487 ± 0.1568 ^cd^	0.7834 ± 0.2355 ^hi^
YC13	Suyu	1.2814 ± 0.0593 ^cd^	1.8227 ± 0.0289 ^i^	1.2058 ± 0.2976 ^bc^	0.9046 ± 0.0431 ^ghi^
YC14	Tongyu 6	1.1700 ± 0.0170 ^fg^	1.1879 ± 0.0102 ^o^	0.3404 ± 0.0405 ^j^	0.5200 ± 0.0623 ^k^
YC15	Tongyu 7	1.0575 ± 0.0299 ^i^	1.7588 ± 0.0288 ^j^	0.4097 ± 0.1529 ^j^	1.1628 ± 0.0262 ^efg^
WC1	Wan	1.2608 ± 0.0186 ^de^	2.1916 ± 0.0157 ^e^	0.4795 ± 0.1540 ^hij^	0.8325 ± 0.1850 ^gh^
WC2	Suke	1.1205 ± 0.0409 ^gh^	1.6802 ± 0.0206 ^k^	1.1135 ± 0.3713 ^efg^	0.7336 ± 0.0984 ^ijk^
WC3	Suyu 1	1.4064 ± 0.0432 ^b^	2.8930 ± 0.0164 ^a^	1.1941 ± 0.1650 ^cde^	1.1017 ± 0.0118 ^fg^
WC4	Huzihei 2	1.3498 ± 0.0457 ^c^	2.8882 ± 0.0319 ^a^	1.9007 ± 0.3514 ^a^	1.5730 ± 0.1009 ^c^
WC5	Huhong 1	1.5803 ± 0.0217 ^a^	2.8194 ± 0.0173 ^b^	0.8301 ± 0.0509 ^fg^	2.1808 ± 0.2335 ^b^
WC6	Sutian	1.0775 ± 0.0287 ^i^	2.3974 ± 0.1078 ^d^	1.0928 ± 0.4205 ^b^	1.0212 ± 0.1923 ^efg^
WC7	Suyu 2	1.3569 ± 0.0105 ^c^	2.6276 ± 0.0358 ^c^	0.6848 ± 0.1496 ^fghi^	0.9384 ± 0.0575 ^ghi^
WC8	Suyuzi	0.9679 ± 0.0147 ^j^	1.6873 ± 0.0256 ^k^	1.2049 ± 0.1201 ^cd^	0.6828 ± 0.1978 ^hij^

Data represent mean values ± standard deviations (n = 3). Different lowercase letters within the same column indicate significant differences between samples (*p* < 0.05).

**Table 3:** Identification of polyphenols in different cultivars of corn extracts.

NO.	T_R_ (min)	Precursor Ion (*m/z*)	Ion Mode	Fragment Ions (*m/z*)	Formula	Identification	Reference
1	0.866	165.0545	[M + H]^+^	95.0497, 77.0395	C_9_H_8_O_3_	3-Hydroxycinnamic acid	[37]
2	0.868	433.1142	[M]^+^	271.061	C_21_H_21_O_10_	Pelargonidin-3-O-glucoside	[38]
3	0.874	449.1081	[M + H]^+^	126.0543	C_21_H_20_O_11_	Luteolin-7-glucoside	[39]
4	5.172	449.1085	[M − 2H]^−^	287.0556	C_21_H_21_O_11_	Cyanidin-3-glucoside	[40]
5	5.838	433.1132	[M + H]^+^	271.0605	C_21_H_20_O_10_	Apigenin-7-O-glucoside	[41]
6	7.886	609.1464	[M − 2H]^−^	300.0273, 271.0256	C_27_H_30_O_16_	Rutin	[42]
7	8.112	463.0878	[M − H]^−^	300.0276, 271.0253	C_21_H_20_O_12_	Isoquercitin	[43]
8	8.972	611.4864	[M]^+^	566.4269, 548.4167	C_27_H_31_O_16_	Cyanidin-3,5-di-O-glucoside chloride	[44]
9	10.075	449.1016	[M + H]^+^	192.9961, 267.0320	C_21_H_20_O_11_	Quercetin 7-rhamnoside	[45]
10	10.739	289.0715	[M + H]^+^	153.0192, 163.0393	C_15_H_12_O_6_	Dihydroluteolin	[46]
11	11.011	303.0513	[M + H]^+^	153.0192, 229.0511	C_15_H_10_O_7_	Quercitrin	[47]
12	12.307	273.0757	[M + H]^+^	153.0190, 147.0445	C_15_H_12_O_5_	Naringenin	[43]
13	12.742	303.0867	[M + H]^+^	153.0190, 177.0553	C_16_H_14_O_6_	Hesperetin	[43]
14	13.681	177.0549	[M + H − H_2_O]^+^	89.0395, 161.0937	C_10_H_10_O_4_	Ferulic acid	[48]
15	13.732	147.0442	[M + H]^+^	65.0394, 91.0542	C_9_H_6_O_2_	Coumarin	[49]
16	16.024	223.0638	[M + H]^+^	191.0014, 207.0325	C_11_H_10_O_5_	Isofraxidin	[50]
17	16.115	163.0402	[M − H]^−^	119.0505, 145.8931	C_9_H_8_O_3_	P-Coumaric acid	[49]
18	16.174	163.0393	[M − H]^−^	119.0501, 148.0112	C_9_H_8_O_3_	Coumaric acid	[51]
19	22.727	577.1344	[M + H]^+^	149.0235, 193.0496	C_30_H_24_O_12_	Procyanidin A1	[52]
20	22.752	577.1341	[M + H]^+^	149.0238, 193.0500	C_30_H_24_O_12_	Procyanidin A2	[52]

**Table 4:** The polyphenol contents in extracts of different cultivars of corn grain.

Sample	Ferulic Acid ^1^	Isofraxidin ^1^	Quercetin 7-Rhamnoside ^2^	Coumarin ^2^	Isosakuranin ^2^	Procyanidin A1 ^2^	Procyanidin A2 ^2^
YC1	333.79 ± 6.04 ^b^	ND	ND	ND	ND	ND	355.66 ± 1.50 ^j^
YC2	195.78 ± 3.62 ^b^	56.75 ± 0.71 ^d^	ND	160.69 ± 2.12 ^a^	ND	ND	346.64 ± 2.97 ^k^
YC3	102.85 ± 4.83 ^f^	ND	ND	ND	ND	601.17 ± 4.18 ^a^	ND
YC4	41.22 ± 1.81 ^h^	69.30 ± 1.41 ^b^	ND	ND	ND	ND	503.44 ± 2.47 ^b^
YC5	97.88 ± 2.41 ^g^	60.83 ± 1.48 ^c^	ND	86.51 ± 1.56 ^e^	ND	ND	300.00 ± 3.82 ^n^
YC6	113.76 ± 6.04 ^e^	89.98 ± 0.49 ^a^	ND	ND	ND	556.42 ± 2.56 ^b^	ND
YC7	ND	ND	65.94 ± 0.57 ^c^	ND	ND	ND	399.82 ± 1.84 ^g^
YC8	ND	ND	90.52 ± 1.09 ^a^	ND	ND	ND	318.44 ± 1.48 ^l^
YC9	ND	51.75 ± 0.99 ^e^	ND	ND	ND	ND	425.64 ± 3.68 ^f^
YC10	ND	ND	76.81 ± 1.84 ^b^	ND	ND	ND	534.88 ± 2.83 ^a^
YC11	ND	41.19 ± 0.57 ^g^	ND	ND	ND	ND	485.28 ± 3.54 ^d^
YC12	ND	43.97 ± 1.13 ^f^	ND	ND	ND	ND	431.21 ± 2.12 ^e^
YC13	ND	40.84 ± 1.27 ^g^	ND	ND	ND	ND	279.70 ± 1.20 ^o^
YC14	ND	38.05 ± 0.49 ^h^	ND	ND	36.72 ± 2.32 ^b^	294.61 ± 1.77 ^d^	ND
YC15	ND	ND	ND	ND	ND	ND	311.78 ± 1.27 ^m^
WC1	ND	44.33 ± 1.48 ^f^	ND	101.30 ± 1.63 ^d^	ND	ND	494.39 ± 2.05 ^c^
WC2	ND	ND	ND	114.91 ± 2.83 ^c^	ND	ND	400.45 ± 4.74 ^g^
WC3	93.07 ± 4.22 ^g^	51.24 ± 0.99 ^e^	ND	100.32 ± 3.31 ^d^	ND	436.67 ± 2.05 ^c^	ND
WC4	144.04 ± 3.02 ^d^	37.66 ± 1.06 ^h^	ND	ND	ND	ND	388.07 ± 3.61 ^h^
WC5	736.98 ± 5.91 ^a^	ND	ND	149.91 ± 3.54 ^b^	ND	ND	368.96 ± 1.48 ^i^
WC6	ND	ND	ND	ND	ND	ND	ND
WC7	ND	ND	ND	ND	ND	ND	ND
WC8	ND	43.16 ± 0.71 ^f^	ND	118.40 ± 2.40 ^c^	58.14 ± 2.16 ^a^	ND	435.52 ± 2.05 ^e^

Data represent mean values ± standard deviations (n = 3). Different lowercase letters within the same column indicate significant differences between samples (*p* < 0.05). ^1^ Quantified as standard (μg/g dw). ^2^ Quantified as Ferulic acid (μg/g dw). ND: not detected.

**Table 5:** The polyphenol contents in extracts of different cultivars of corn husk.

Sample	Naringenin ^1^	Ferulic Acid ^1^	Isofraxidin ^1^	Coumarin ^2^	Coumaric Acid ^2^	Procyanidin A1 ^2^	Procyanidin A2 ^2^
YC1	ND	72.18 ± 1.31 ^e^	ND	ND	ND	473.14 ± 3.43 ^a^	ND
YC2	ND	118.85 ± 2.93 ^d^	38.69 ± 0.93 ^g^	208.47 ± 1.74 ^a^	ND	ND	210.24 ± 1.95 ^j^
YC3	ND	ND	ND	ND	ND	ND	384.48 ± 1.08 ^a^
YC4	ND	ND	55.50 ± 1.06 ^c^	ND	ND	211.81 ± 1.55 ^f^	ND
YC5	ND	ND	ND	ND	ND	ND	237.50 ± 1.77 ^h^
YC6	13.62 ± 1.47 ^b^	ND	70.52 ± 3.17 ^b^	ND	49.00 ± 1.42 ^c^	ND	218.19 ± 3.40 ^i^
YC7	ND	ND	ND	ND	ND	ND	295.79 ± 0.86 ^d^
YC8	ND	ND	39.69 ± 0.22 ^fg^	ND	ND	399.90 ± 2.12 ^b^	ND
YC9	ND	ND	48.48 ± 1.76 ^d^	ND	64.14 ± 2.73 ^b^	ND	135.88 ± 0.80 ^l^
YC10	ND	ND	ND	ND	ND	ND	351.52 ± 1.75 ^c^
YC11	ND	ND	31.02 ± 0.69 ^hi^	ND	75.76 ± 2.29 ^a^	ND	376.09 ± 2.06 ^b^
YC12	ND	ND	43.53 ± 1.04 ^de^	ND	ND	ND	268.47 ± 1.79 ^e^
YC13	ND	ND	32.38 ± 1.68 ^hi^	ND	51.68 ± 0.93 ^c^	ND	245.78 ± 0.55 ^g^
YC14	1.19 ± 0.08 ^c^	ND	41.00 ± 3.53 ^def^	ND	ND	ND	138.53 ± 2.50 ^l^
YC15	ND	ND	39.06 ± 2.08 ^efg^	ND	ND	ND	390.21 ± 2.27 ^a^
WC1	ND	ND	ND	85.78 ± 0.86 ^f^	39.25 ± 1.94 ^d^	241.31 ± 2.61 ^e^	ND
WC2	ND	ND	33.98 ± 0.72 ^h^	79.29 ± 1.92 ^g^	ND	252.80 ± 1.98 ^d^	ND
WC3	ND	ND	ND	127.40 ± 1.70 ^d^	ND	ND	221.74 ± 1.23 ^i^
WC4	76.34 ± 0.74 ^a^	352.81 ± 2.26 ^c^	ND	70.03 ± 1.39 ^h^	ND	ND	261.78 ± 2.67 ^f^
WC5	ND	1101.90 ± 12.80 ^a^	75.58 ± 3.12 ^a^	175.84 ± 2.94 ^c^	ND	ND	270.24 ± 2.29 ^e^
WC6	ND	427.60 ± 1.70 ^b^	30.10 ± 1.49 ^i^	188.41 ± 1.13 ^b^	ND	ND	240.19 ± 1.28 ^h^
WC7	ND	ND	41.04 ± 1.38 ^efg^	116.95 ± 0.74 ^e^	ND	ND	162.34 ± 1.88 ^k^
WC8	ND	ND	ND	ND	ND	311.53 ± 1.04 ^c^	ND

Data represent mean values ± standard deviations (n = 3). Different lowercase letters within the same column indicate significant differences between samples (*p* < 0.05). ^1^ Quantified as standard (μg/g dw). ^2^ Quantified as Ferulic acid (μg/g dw). ND: not detected.

**Table 6:** Antioxidant activity of different corn extracts.

NO.	Sample	DPPH (μM TE/g dw)		ABTS (μM TE/g dw)		FRAP (μM TE/g dw)	
		Corn Grain	Corn Husk	Corn Grain	Corn Husk	Corn Grain	Corn Husk
YC1	Sukeyu 1	1.82 ± 0.42 ^de^	1.35 ± 0.06 ^def^	7.01 ± 2.14 ^bcde^	10.99 ± 1.65 ^j^	2.97 ± 0.69 ^cdef^	2.21 ± 0.02 ^hi^
YC2	Sukeyu 2	1.49 ± 0.03 ^hij^	1.44 ± 0.06 ^def^	5.84 ± 0.92 ^cde^	16.24 ± 1.87 ^de^	2.22 ± 0.52 ^def^	2.84 ± 0.08 ^ghi^
YC3	Huai	1.34 ± 0.01 ^jk^	1.30 ± 0.03 ^efg^	7.13 ± 4.09 ^bcde^	16.57 ± 1.23 ^de^	2.09 ± 0.23 ^def^	2.89 ± 0.36 ^ghi^
YC4	Jiangyu 1	1.06 ± 0.27 ^m^	0.76 ± 0.07 ^kl^	5.59 ± 1.53 ^cde^	10.48 ± 0.52 ^j^	2.2 ± 0.45 ^def^	1.92 ± 0.27 ^i^
YC5	Jiangyu 2	1.62 ± 0.07 ^fgh^	1.28 ± 0.67 ^efg^	5.92 ± 1.9 ^cde^	17.91 ± 0.94 ^cd^	2.28 ± 0.63 ^cdef^	3.44 ± 0.25 ^fgh^
YC6	Zhongjiang 1	1.20 ± 0.02 ^klm^	1.05 ± 0.10 ^ghij^	6.70 ± 0.37 ^bcde^	12.64 ± 0.48 ^ghij^	1.66 ± 0.65 ^f^	2.92 ± 0.12 ^ghi^
YC7	Zhongjiang 2	1.71 ± 0.01 ^ef^	1.23 ± 0.06 ^fgh^	5.43 ± 1.39 ^cde^	19.03 ± 1.78 ^c^	2.55 ± 0.63 ^cdef^	2.34 ± 0.07 ^hi^
YC8	Tongyu 1	1.49 ± 0.05 ^hij^	1.42 ± 0.10 ^def^	6.47 ± 0.20 ^cde^	14.92 ± 0.94 ^efg^	2.37 ± 1.38 ^cdef^	5.05 ± 2.32 ^cd^
YC9	Tongyu 2	1.44 ± 0.04 ^ij^	1.61 ± 0.06 ^cd^	5.21 ± 2.00 ^cde^	15.60 ± 1.16 ^ef^	2.38 ± 0.37 ^cdef^	3.77 ± 0.40 ^efg^
YC10	Tongyu 3	1.19 ± 0.12 ^klm^	0.96 ± 0.11 ^ijkl^	3.83 ± 0.77 ^e^	14.48 ± 0.63 ^efgh^	1.69 ± 0.63 ^f^	2.24 ± 0.05 ^hi^
YC11	Tongyu 4	1.11 ± 0.19 ^lm^	1.27 ± 0.06 ^efg^	5.54 ± 1.05 ^cde^	14.36 ± 1.69 ^efgh^	1.81 ± 0.93 ^ef^	4.03 ± 0.54 ^defg^
YC12	Tongyu 5	1.25 ± 0.04 ^kl^	0.85 ± 0.11 ^jkl^	4.28 ± 1.32 ^de^	13.71 ± 2.32 ^fghi^	1.84 ± 0.12 ^ef^	2.46 ± 0.22 ^hi^
YC13	Suyu	2.00 ± 0.04 ^c^	1.87 ± 0.05 ^b^	6.22 ± 1.23 ^cde^	15.74 ± 0.67 ^def^	3.8 ± 0.24 ^bc^	3.38 ± 0.32 ^fgh^
YC14	Tongyu 6	1.60 ± 0.09 ^fghi^	0.90 ± 0.02 ^jkl^	13.57 ± 6.45 ^a^	11.02 ± 0.26 ^j^	3.82 ± 0.24 ^bc^	3.13 ± 0.62 ^ghi^
YC15	Tongyu 7	1.67 ± 0.32 ^efg^	1.00 ± 0.07 ^hijk^	7.57 ± 1.15 ^bcd^	14.65 ± 1.77 ^efg^	3.43 ± 0.52 ^bcd^	4.38 ± 0.64 ^def^
WC1	Wan	1.53 ± 0.15 ^ghi^	1.38 ± 0.08 ^def^	8.19 ± 1.94 ^bc^	15.69 ± 0.76 ^def^	3.29 ± 0.34 ^bcde^	3.87 ± 0.30 ^defg^
WC2	Suke	1.65 ± 0.20 ^efgh^	1.38 ± 0.07 ^jkl^	5.31 ± 0.31 ^cde^	12.00 ± 1.06 ^ij^	3.32 ± 0.79 ^bcde^	3.44 ± 0.69 ^fgh^
WC3	Suyu 1	1.92 ± 0.07 ^cd^	1.84 ± 0.09 ^bc^	7.38 ± 1.61 ^bcd^	16.63 ± 2.06 ^de^	1.91 ± 1.21 ^def^	5.10 ± 0.31 ^cd^
WC4	Huzihei 2	3.01 ± 0.06 ^a^	2.30 ± 0.06 ^a^	9.88 ± 1.09 ^b^	25.99 ± 1.97 ^a^	5.53 ± 1.31 ^a^	5.95 ± 0.86 ^bc^
WC5	Huhong 1	2.72 ± 0.01 ^b^	2.32 ± 0.06 ^a^	8.40 ± 0.29 ^bc^	26.57 ± 2.03 ^a^	4.71 ± 2.24 ^ab^	8.12 ± 1.34 ^a^
WC6	Sutian	1.33 ± 0.05 ^jk^	1.49 ± 0.06 ^de^	6.58 ± 0.80 ^bcde^	21.66 ± 1.71 ^b^	2.98 ± 0.12 ^cdef^	6.89 ± 1.09 ^ab^
WC7	Suyu 2	1.89 ± 0.08 ^cd^	1.19 ± 0.06 ^fghi^	8.29 ± 2.33 ^bc^	19.84 ± 0.97 ^bc^	3.04 ± 1.73 ^cdef^	4.85 ± 0.36 ^cde^
WC8	Suyuzi	1.14 ± 0.01 ^lm^	0.72 ± 0.01 ^l^	5.75 ± 0.49 ^cde^	12.24 ± 0.26 ^hij^	3.82 ± 1.64 ^bc^	3.19 ± 1.20 ^fgh^

Data represent mean values ± standard deviations (n = 3). Different lowercase letters within the same column indicate significant differences between samples (*p* < 0.05).

**Table 7:** Correlation analysis of compounds and antioxidant activity.

		DPPH		ABTS		FRAP	
	Compounds	Corn Grain	Corn Husk	Corn Grain	Corn Husk	Corn Grain	Corn Husk
Yellow corn	lutein	−0.4061	0.3911	−0.3927	0.5246	−0.6102	0.4535
	TPC	0.5670	0.4593	0.1353	0.3996	0.5328	0.3791
	TFC	0.1786	0.5364	−0.6198	0.3296	−0.2220	0.4472
Waxy corn	lutein	0.6844	0.5290	0.6375	0.5105	0.7064	0.1806
	TPC	0.7912	0.8727	0.7253	0.8685	0.2041	0.7258
	TFC	0.4187	0.8911	0.1488	0.8685	0.4553	0.8602

## Data Availability

The original contributions presented in the study are included in the article, further inquiries can be directed to the corresponding author.
